# The IgG Paradox in Lipedema: More Food Sensitivities, Less Antibody Production

**DOI:** 10.7759/cureus.93788

**Published:** 2025-10-03

**Authors:** Alexandre C Amato, Juliana S Amato, Daniel Benitti

**Affiliations:** 1 Department of Vascular Surgery, Amato - Instituto de Medicina Avançada, São Paulo, BRA; 2 Department of Gynecology, Amato Instituto de Medicina Avançada, São Paulo, BRA; 3 Department of Vascular and Endovascular Surgery, Medical Valens Center, São Paulo, BRA

**Keywords:** complement system, dietary diversity, food reactivity, food sensitivity, igg, immune dysregulation, intestinal permeability, lipedema

## Abstract

Background

Lipedema is a chronic adipose tissue disorder affecting primarily women and is increasingly associated with immune dysregulation and intestinal permeability. Food-specific IgG testing has been explored in various inflammatory conditions, but its relevance to lipedema remains unknown.

Objective

The objective of this study is to characterize IgG food sensitivity profiles in women with lipedema and investigate the paradoxical relationship between increased food reactivity and reduced total IgG antibody levels.

Methods

We conducted a retrospective cross-sectional study involving 234 participants: women with lipedema (n=80), women without lipedema (n=74), and men (n=80). All had undergone IgG testing against 222 food antigens via ELISA. We analyzed qualitative (positive/negative) and quantitative IgG reactivity, applied dimensionality reduction (PCA, t-SNE) and clustering, and developed a multivariable logistic regression model to assess diagnostic performance.

Results

Women with lipedema exhibited a non-significantly higher number of positive IgG food reactions (14.8 vs 12.6; *p*=0.186), despite significantly lower total IgG levels (1747.1 vs 2974.8 AU; *p*<0.001). This paradox was consistent across 79.7% of tested antigens. The most discriminative foods included wild game meats and certain vegetables. A combined IgG-based model achieved an area under the curve of 0.804, outperforming individual IgG metrics. Dimensionality reduction revealed no clear clustering based on reactivity patterns alone.

Conclusion

Lipedema displays a paradoxical IgG signature, more frequent positives despite lower total IgG, consistent with mucosal immune dysregulation (e.g., increased intestinal permeability, immune exhaustion, or dietary monotony). Single IgG metrics had limited discrimination, but a combined score improved classification, supporting IgG profiling as a complementary, not standalone, biomarker for patient stratification and personalized dietary guidance. Collectively, these findings suggest that the adipose phenotype may be downstream of broader systemic processes; prospective studies should assess IgG subclasses, barrier markers (e.g., zonulin), and gluten-modulated interventions.

## Introduction

Lipedema is a chronic adipose tissue disorder characterized by the symmetric, disproportionate accumulation of subcutaneous fat in the lower extremities, affecting women almost exclusively, with an estimated prevalence of 12.3% [1]. Despite its high prevalence, lipedema remains poorly understood and frequently misdiagnosed, leading to inadequate treatment and a significant impact on quality of life [2,3]. Growing evidence points to immune dysfunction, metabolic dysregulation, and chronic low-grade inflammation as central to its pathophysiology [4-7].

The inflammatory state in lipedema appears fundamentally different from that in obesity alone, with altered cytokine profiles and dysregulation of immune responses, including a significant downregulation of complement factor D (CFD) [4]. In this context, food-specific IgG may function as a quantitative readout of mucosal antigen exposure and tolerance in disorders with suspected barrier dysfunction, such as lipedema.

Food-specific IgG antibodies have generated significant debate regarding their role in immune tolerance versus food intolerance [8]. These antibodies, particularly those of the IgG4 subclass, are frequently detected in individuals without adverse food reactions and may represent a normal adaptive immune response to constant dietary antigen exposure, contributing to the development of regulatory T-cell-mediated tolerance [8]. However, there is growing interest in their potential role in chronic inflammation and immune activation, particularly in diseases marked by impaired intestinal barrier function. Elevated levels of food-specific IgG have been observed in eosinophilic esophagitis, irritable bowel syndrome, inflammatory bowel disease, and autoimmune conditions [8-14].

The diagnostic utility of food-specific IgG testing remains controversial. However, multiple investigations have demonstrated that IgG-guided elimination diets can yield symptomatic improvements in various conditions, suggesting potential benefits for targeted dietary interventions [8-15].

Emerging evidence suggests a link between intestinal permeability, food antigen reactivity, and lipedema pathophysiology. We previously demonstrated increased prevalence of HLA-DQ2 (47.4%) and HLA-DQ8 (22.2%) genotypes in symptomatic lipedema patients compared to controls, suggesting a potential connection with gluten sensitivity [7]. These HLA genotypes are associated with increased intestinal permeability, which can facilitate the translocation of bacterial lipopolysaccharides (LPSs) and food antigens, thereby triggering systemic inflammation [16,17]. Elevated levels of specific IgG antibodies against food antigens are frequently documented in conditions that exhibit increased intestinal barrier permeability, particularly in celiac disease, inflammatory bowel disease, and immunoglobulin A deficiency [8].

IgG responses are typically part of physiological immune clearance [12]. However, when produced in excess, these antibodies may contribute to immune complex deposition and tissue inflammation, particularly in the gut and vasculature [12].

Food-specific IgG testing may identify delayed-type responses associated with low-grade inflammation and increased intestinal permeability [18]. In lipedema, where immune dysregulation is a central feature, understanding IgG food sensitivity patterns could provide insights into disease mechanisms.

This study aimed to comprehensively characterize IgG food sensitivity profiles in women with lipedema, comparing both qualitative and quantitative patterns to matched controls across 222 food antigens. We hypothesized that lipedema patients would exhibit distinct food sensitivity patterns contributing to their chronic inflammatory state. We also explored the apparent paradox of increased antigen reactivity alongside altered total IgG output, suggesting a unique immune signature in this population. Clarifying this relationship could help guide personalized dietary strategies and improve the clinical management of lipedema.

## Materials and methods

Study design and participants

This retrospective cross-sectional study analyzed IgG food sensitivity profiles in 234 participants, divided into three groups: women with lipedema (n=80), women without lipedema (n=74), and men (n=80). Participants were recruited through convenience sampling from patients attending outpatient visits at a clinical center who had undergone comprehensive IgG antibody testing against 222 food antigens using a commercial ELISA-based food sensitivity panel between January 2020 and December 2024.

The diagnosis of lipedema was confirmed by clinical examination according to established diagnostic criteria [19]. Control groups consisted of individuals without a diagnosis of lipedema who underwent the same IgG testing during the same period.

Inclusion criteria were age ≥18 years and availability of complete test data. Exclusion criteria regarding inflammatory or autoimmune conditions and the use of immunomodulatory medications were deliberately not applied, in order to reflect the real-world heterogeneity of individuals undergoing IgG food sensitivity testing.

This study was approved by the Research Ethics Committee under CAAE 90941025.3.0000.0081, registered in Plataforma Brasil, which waived the requirement for individual informed consent due to the retrospective nature of the study and the anonymization of all data.

Laboratory analysis

Blood samples were collected following an overnight fast. Food-specific IgG was measured using a microarray-based enzyme immunoassay with HRP/TMB colorimetric detection (FoodPrint 200+ IgG microarray; Omega Diagnostics Ltd., Eden Research Park, Cambridgeshire, UK; catalog CSN301-4x4), covering 222 food antigens. Briefly, patient serum was incubated for 30 minutes at room temperature on glass slides printed with food extracts; unbound material was removed by washing, followed by incubation with anti-human IgG conjugated to horseradish peroxidase (HRP) for a further 30 minutes at room temperature. After additional washes, 3,3′,5,5′-tetramethylbenzidine (TMB) substrate was applied for 10 minutes; slides were then rinsed with distilled water, dried by centrifugation, and scanned on a high-resolution flatbed scanner. The manufacturer’s software converted spot optical densities to quantitative readouts. Positivity was defined as ≥30 U/mL per the manufacturer’s validated cutoff; borderline results (e.g., 24-30 U/mL) were handled per manufacturer guidance; binary outcomes followed this rule. For continuous analyses (e.g., total and mean IgG signal), we used the manufacturer-normalized arbitrary units (AU) exported by the software. All assays were performed in the same accredited laboratory under the manufacturer’s SOPs.

Statistical analysis

Primary group comparisons were performed using Student’s t-test for continuous variables and the chi-square test for categorical variables. Effect sizes were calculated using Cohen’s d to estimate the magnitude of differences across food antigens. The diagnostic performance of IgG parameters was assessed using receiver operating characteristic (ROC) curves, with area under the curve (AUC) values reported. To account for multiple comparisons, the Benjamini-Hochberg correction was applied where appropriate.

Exploratory analyses included Pearson correlation matrices to evaluate co-reactivity among foods. Analyses used complete-case data per antigen; per-food denominators are reported in tables. Dimensionality reduction techniques, such as principal component analysis (PCA) and t-distributed stochastic neighbor embedding (t-SNE), were applied for pattern visualization. T-SNE is a method that helps visualize patterns in complex data by mapping it into a two-dimensional plot-essentially showing how similar or different patients are based on multiple IgG measurements. Hierarchical clustering was performed using Ward’s method with Euclidean distance to identify natural groupings among participants. Classifier performance used stratified 5-fold cross-validation repeated 20 times with different random seeds; mean AUC and SD are reported.

Finally, a multivariable logistic regression model was developed to assess the combined diagnostic potential of selected IgG parameters.

All analyses were conducted using Python 3.9, with the following libraries: pandas, numpy, scipy, scikit-learn, and matplotlib. A two-tailed p-value < 0.05 was considered statistically significant.

## Results

Participant characteristics

A total of 234 individuals were included (women with lipedema, n=80; women without lipedema, n=74; men, n=80). Groups were comparable in the testing window. Demographic summary is shown in Table 1.

**Table 1:** Demographic characteristics of the sample Per-food sample sizes may vary slightly due to missing values; analyses used all available cases. IQR: interquartile range; SD: standard deviation.

Group	n	Age, Mean ± SD (y)	Age, Median (IQR)	Sex (F/M)
Women with lipedema	80	48.0 ± 12.1	49 (40–57)	80/0
Women without lipedema	74	47.0 ± 11.3	47 (39–56)	74/0
Men	80	46.5 ± 12.9	47 (38–56)	0/80

Comparative analyses focused on women only due to sex-based immunological differences. Subgroup analysis by reactivity tertiles is presented in Table 2, highlighting the significant difference within the low-reactivity stratum.

**Table 2:** Subgroup analysis of positive IgG food tests by reactivity tertiles Values are mean ± SD of the number of positive IgG tests; comparisons between women with lipedema and women without lipedema were performed within tertiles using two-tailed Welch’s t-tests (unequal variances). Statistics: Low (0–8) t = −2.19, df ≈ 47.9, p = 0.040*; Medium (9–16) t = −0.56, df ≈ 54.0, p = 0.597; High (≥17) t = 1.00, df ≈ 32.9, p = 0.404. Overall (all women): 14.8 ± 12.2 (lipedema, n=80) vs 12.6 ± 7.5 (controls, n=74); Welch’s t = 1.359, df ≈ 132.76, p = 0.176. *p < 0.05.

Tertile (Range)	Without Lipedema	With Lipedema	p-value
Low (0-8 tests)	n=28, 5.6 ± 2.0	n=23, 4.4 ± 1.9	0.040*
Medium (9-16 tests)	n=27, 12.2 ± 1.9	n=29, 11.9 ± 2.1	0.597
High (≥17 tests)	n=19, 23.5 ± 3.9	n=28, 26.3 ± 14.0	0.404

While the overall number of positive tests did not differ significantly between groups, exploratory subgroup analysis revealed significant differences in the low-reactivity subset (≤8 positive foods; p=0.040). This finding, combined with the highly significant differences in total IgG levels (p<0.001), suggests a complex immunological pattern in lipedema characterized by altered antibody production rather than simply increased food reactivity. Participant characteristics and summary IgG metrics are summarized in Table 3.

**Table 3:** Participant characteristics and summary IgG metrics by group Group-level descriptive statistics for qualitative (number of positive tests) and quantitative IgG measures in women with lipedema, women without lipedema, and men. Between-group comparisons for women used two-tailed Welch’s t-tests (unequal variances) and are reported as t(df) and two-sided p: Total IgG t = −6.949, df ≈ 150.64, p = 1.01×10⁻¹⁰; Mean IgG per food same t/df/p (linear rescale of Total IgG); Maximum IgG t ≈ −0.57, df ≈ 124.2, p ≈ 0.342; IgG per positive test t ≈ −4.47, df ≈ 88.5, p < 0.001. Women with lipedema showed lower Total IgG (mean 1747.1 ± 1086.7 AU vs 2974.8 ± 1104.9 AU), lower Mean IgG per food (7.9 ± 4.9 vs 13.4 ± 5.0), and lower IgG per positive test (118.1 ± 73.4 vs 236.1 ± 215.9), while Maximum IgG did not differ significantly. Medians with IQRs and observed ranges are provided for distributional context. Values are mean ± SD unless otherwise indicated. ^a^ p-value for comparison between women with and without lipedema (independent t-test; men shown descriptively). ^b^ Average IgG intensity among tests classified as positive. IQR: interquartile range; SD: standard deviation.

Characteristic	Women with Lipedema (n=80)	Women without Lipedema (n=74)	Men (n=80)	p-value ^a^
Qualitative Analysis				
Positive tests, mean ± SD	14.8 ± 12.2	12.6 ± 7.5	17.9 ± 11.4	0.186
Positive tests, median (IQR)	12 (7-19)	11 (7-17)	15 (10-23)	-
Range	1-73	1-31	1-52	-
Quantitative Analysis				
Total IgG, mean ± SD	1747.1 ± 1086.7	2974.8 ± 1104.9	2565.7 ± 1216.5	<0.001
Total IgG, median (IQR)	1530 (980-2120)	3484 (1981-3788)	2315 (1619-3718)	-
Mean IgG per food	7.9 ± 4.9	13.4 ± 5.0	11.6 ± 5.5	<0.001
Maximum IgG value	84.7 ± 67.2	89.6 ± 36.7	97.1 ± 48.9	0.342
IgG per positive test^b^	118.1 ± 73.4	236.1 ± 215.9	143.3 ± 106.7	<0.001

Qualitative analysis of IgG reactivity

**Figure 1:**
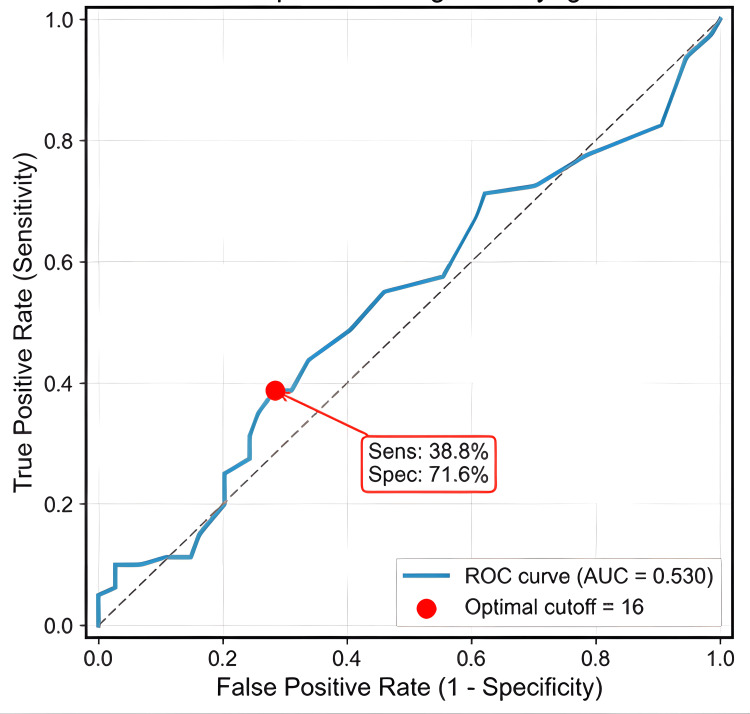
ROC curve for lipedema diagnosis using the number of positive IgG food tests Receiver operating characteristic (ROC) curve using the count of positive IgG food tests as the predictor. AUC = 0.530, indicating modest discrimination. The red marker denotes the Youden-optimal cutoff of 16 positive tests (sensitivity = 38.8%, specificity = 71.6%). The dashed diagonal is the no-discrimination reference line (AUC = 0.5); axes show true positive rate (sensitivity) versus false positive rate (1 − specificity).

Women with lipedema showed a non-significant trend toward a higher mean number of positive food reactions compared to women without lipedema (14.8 ± 12.2 vs 12.6 ± 7.5, p = 0.186, Cohen's d = 0.214). The optimal diagnostic cutoff of ≥16 positive tests yielded a sensitivity of 38.8% and specificity of 71.6%, with an AUC of 0.530 (95% CI: 0.447-0.613). The ROC curve for the number of positive IgG tests is shown in Figure 1.

**Figure 2:**
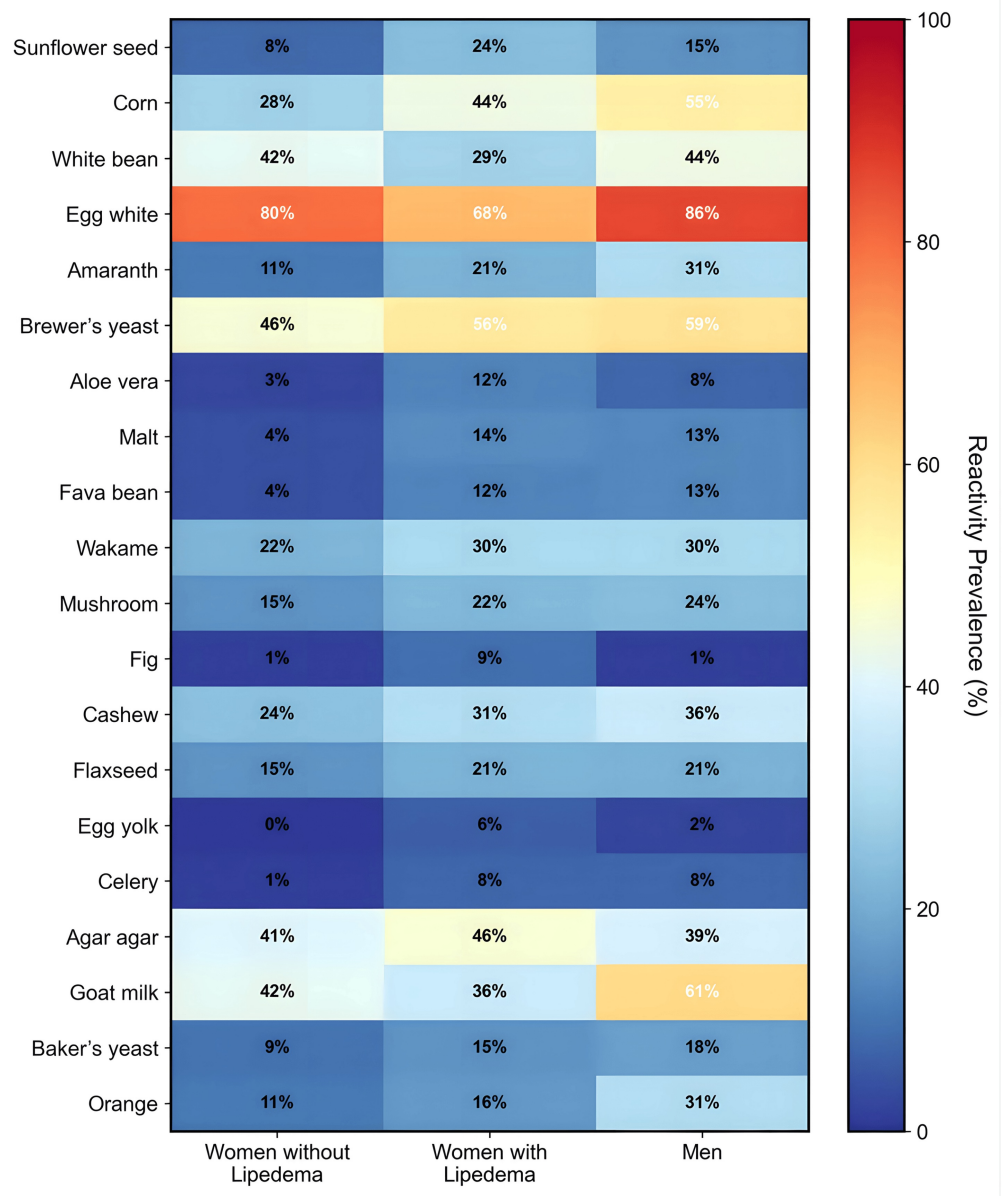
Prevalence of food-specific IgG reactivity for the 20 most discriminative foods Heatmap of the percentage of participants with a positive IgG test for each of the top 20 discriminative foods across groups (women without lipedema, women with lipedema, men). Rows list foods; columns list groups. The color scale indicates prevalence from dark blue (low) to red (high). Notable contrasts include egg white (high across all groups, especially men 86% and women without lipedema 80%) and higher prevalence in women with lipedema for sunflower seed (24% vs 8%) and aloe vera (12% vs 3%) relative to controls, suggesting group-associated immune reactivity patterns.

The most prevalent food reactivities across all groups were barley with 93.8% in lipedema patients, 93.2% in control women, and 98.8% in men; pea with 90.0%, 85.1%, and 95.0% respectively; egg white with 67.5%, 79.7%, and 86.3%; and cow milk with 76.3%, 78.4%, and 82.5%. Two foods showed statistically significant differences between women with and without lipedema: sunflower seed (23.8% vs 8.1%, p = 0.02) and aloe vera (12.5% vs 2.7%, p = 0.05). Patterns for the 20 most discriminative foods across groups are visualized in Figure 2. A ranked list of foods by prevalence of positive IgG tests in each group is provided in Table 4. Foods showing statistically significant differences between women with and without lipedema are detailed in Table 5 and Table 6.

**Table 4:** Top 30 foods by prevalence of positive IgG tests (by group) For each group (women with lipedema, women without lipedema, men), foods are ranked by the percentage of participants with a positive IgG test for that antigen (prevalence). Values are percentages. Rankings are within group. * Indicates a statistically significant difference versus women without lipedema (p < 0.05).

Rank	Women with Lipedema		Women without Lipedema		Men	
	Food	% Positive	Food	% Positive	Food	% Positive
1	Barley	93.8	Barley	93.2	Barley	98.8
2	Pea	90.0	Pea	85.1	Pea	95.0
3	Cow milk	76.3	Egg white	79.7	Egg white	86.3
4	Kola nut	71.3	Cow milk	78.4	Kola nut	85.7
5	Egg white	67.5	Kola nut	73.0	Sheep milk	83.8
6	Sheep milk	62.5	Sheep milk	66.2	Cow milk	82.5
7	Brewer's yeast	56.3	Casein	59.5	Casein	68.8
8	Casein	55.0	Brewer's yeast	45.9	Goat milk	61.3
9	Agar agar	46.3	Goat milk	41.9	Brewer's yeast	58.8
10	Brazil nut	45.0	White bean	41.9	Plum	57.5
11	Corn	43.8	Brazil nut	40.5	Corn	55.0
12	Plum	38.8	Agar agar	40.5	Soybean	55.0
13	Goat milk	36.3	Plum	40.5	White bean	43.8
14	Ginkgo biloba	35.0	Ginkgo biloba	32.4	Brazil nut	42.5
15	Cashew	31.3	Soybean	29.7	Red bean	41.3
16	Wakame	30.0	Corn	28.4	Agar agar	39.0
17	White bean	28.8	Pistachio	28.4	Cashew	36.3
18	Soybean	25.0	Cashew	24.3	Wheat	33.8
19	Pistachio	25.0	Potato	21.6	Pistachio	33.8
20	Wheat	23.8	Wakame	21.6	Potato	33.8
21	Sunflower seed	23.8*	Wheat	18.9	Hazelnut	32.5
22	Mushroom	22.5	Red bean	18.9	Orange	31.3
23	Flaxseed	21.3	Almond	16.2	Amaranth	31.2
24	Amaranth	21.3	Hazelnut	16.2	Wakame	29.9
25	Red bean	18.8	Flaxseed	14.9	Peanut	26.3
26	Potato	18.8	Mushroom	14.9	Ginkgo biloba	24.7
27	Hazelnut	17.5	Oat	13.5	Mushroom	23.8
28	Orange	16.3	Gliadin	12.2	Almond	22.5
29	Baker's yeast	15.0	Peanut	12.2	Flaxseed	20.8
30	Malt	13.8	Amaranth	10.8	Gliadin	20.0

**Table 5:** Qualitative (binary) differences in IgG positivity between women with and without lipedema Proportion of positive IgG results by food antigen shown as % (n/N). Absolute difference: lipedema − control. Prevalence ratio: (lipedema prevalence)/(control prevalence). p-values from two-sided Pearson’s χ²; Fisher’s exact reported when expected counts <5. df: degrees of freedom. “—”: not estimable when control prevalence = 0%. Sample sizes: lipedema n = 80; controls n = 74. Percentages may not total due to rounding; no multiplicity adjustment applied.

Food	Lipedema % (n/N)	Controls % (n/N)	Absolute Difference (%)	Prevalence Ratio	χ² (df=1)	p (χ²)	p (Fisher)
Sunflower seed	23.8 (19/80)	8.1 (6/74)	15.6	2.93	6.92	0.0085	0.0092
Aloe vera	12.5 (10/80)	2.7 (2/74)	9.8	4.62	5.14	0.0234	0.033
Malt	13.8 (11/80)	4.1 (3/74)	9.7	3.39	4.37	0.0365	0.0489
Fig	8.8 (7/80)	1.4 (1/74)	7.4	6.48	4.27	0.0387	0.0649
Egg yolk	6.3 (5/80)	0.0 (0/74)	6.3	—	4.78	0.0288	0.0594

**Table 6:** Quantitative differences (IgG levels) between women with and without lipedema Means ± SD (AU), effect size (Cohen’s d), fold-change (lipedema/control), and p (Welch). Values in arbitrary units (AU). A negative d indicates lower levels in the lipedema group. Two-tailed Welch’s t-tests. SD, standard deviation.

Food	Women with Lipedema (Mean ± SD, AU)	Women without Lipedema (Mean ± SD, AU)	Cohen's d	Fold Change (L/C)	p-value (Welch)
Wild boar	1.8 ± 4.0	11.0 ± 6.1	-1.805	0.17	<0.001***
Shallot	3.7 ± 3.8	11.4 ± 5.3	-1.694	0.32	<0.001***
Rabbit	3.9 ± 4.2	11.6 ± 4.9	-1.685	0.34	<0.001***
Green bean	2.8 ± 3.7	10.7 ± 5.9	-1.613	0.26	<0.001***
Fennel	1.9 ± 3.2	9.8 ± 6.5	-1.585	0.19	<0.001***
Horse	3.7 ± 3.1	10.8 ± 5.6	-1.575	0.34	<0.001***
Veal	1.1 ± 3.1	9.8 ± 7.5	-1.544	0.11	<0.001***
Barnacle	3.2 ± 3.4	10.5 ± 5.8	-1.542	0.31	<0.001***
Cauliflower	2.7 ± 3.5	10.4 ± 6.2	-1.534	0.26	<0.001***
Razor clam	2.4 ± 3.0	9.8 ± 6.4	-1.500	0.24	<0.001***

Quantitative IgG analysis

**Figure 3:**
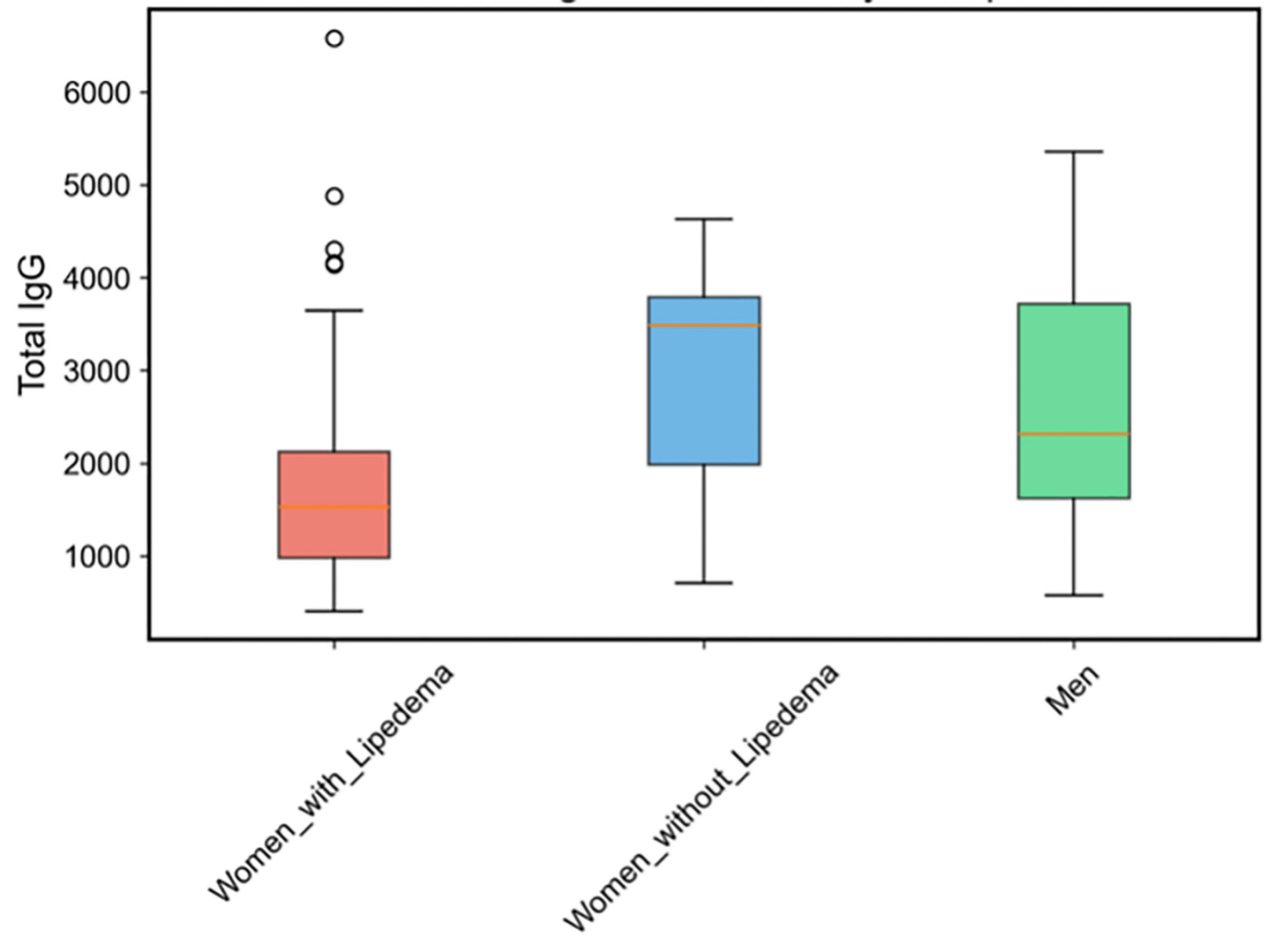
Total IgG distribution by group Box plot of total IgG reactivity (arbitrary units, AU) for women with lipedema (red), women without lipedema (blue), and men (green). Women with lipedema show lower total IgG (median ≈ 1,530 AU) compared with women without lipedema (median ≈ 3,484 AU) and men (median ≈ 2,315 AU). Boxes indicate the interquartile range (IQR) with the median as a horizontal line; whiskers denote 1.5×IQR; outliers are plotted as individual points.

Despite having more positive tests, women with lipedema showed significantly lower total IgG levels (1747.1 ± 1086.7 vs 2974.8 ± 1104.9, p = 1.01 × 10⁻¹⁰). The distribution of total IgG levels across groups is depicted in Figure 3.

**Figure 4:**
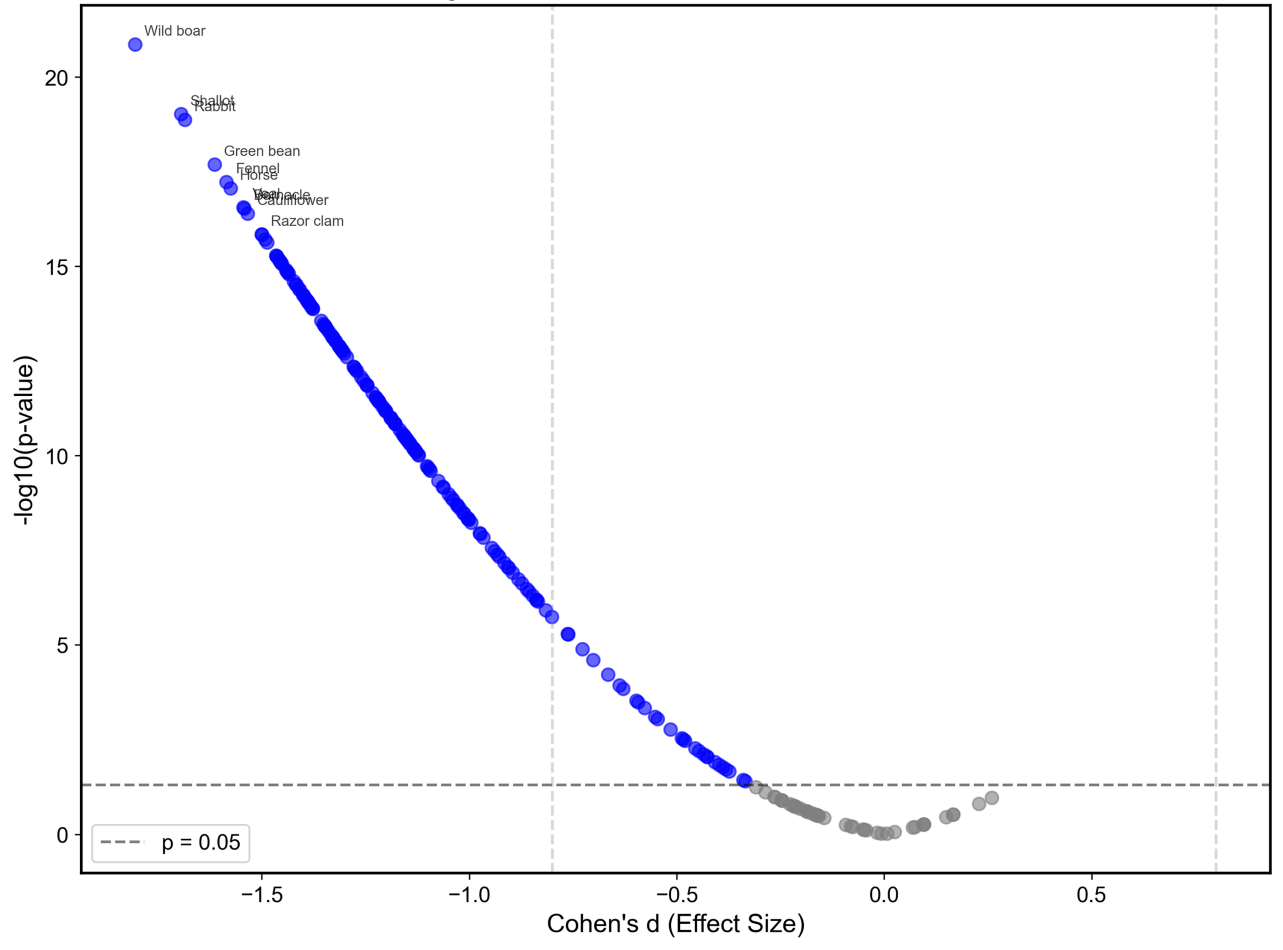
Volcano plot of quantitative IgG differences between women with lipedema and controls Each dot represents one food antigen. The x-axis shows Cohen’s d (negative = lower IgG in lipedema; positive = higher), and the y-axis shows −log10(p-value). Blue points indicate antigens with lower IgG in lipedema (red would indicate higher). The horizontal dashed line marks p = 0.05 (−log10 ≈ 1.30); vertical dashed lines mark |d| = 0.75 (approximate large-effect threshold). Labeled examples (e.g., wild boar, shallot, rabbit, green bean, fennel, sunflower, razor clam) denote the largest effects.

This pattern was consistent across individual foods, with 177/222 (79.7%) foods showing significantly lower IgG concentrations in the lipedema group. Quantitative differences across the 222 antigens are summarized in the volcano plot (Figure 4).

**Figure 5:**
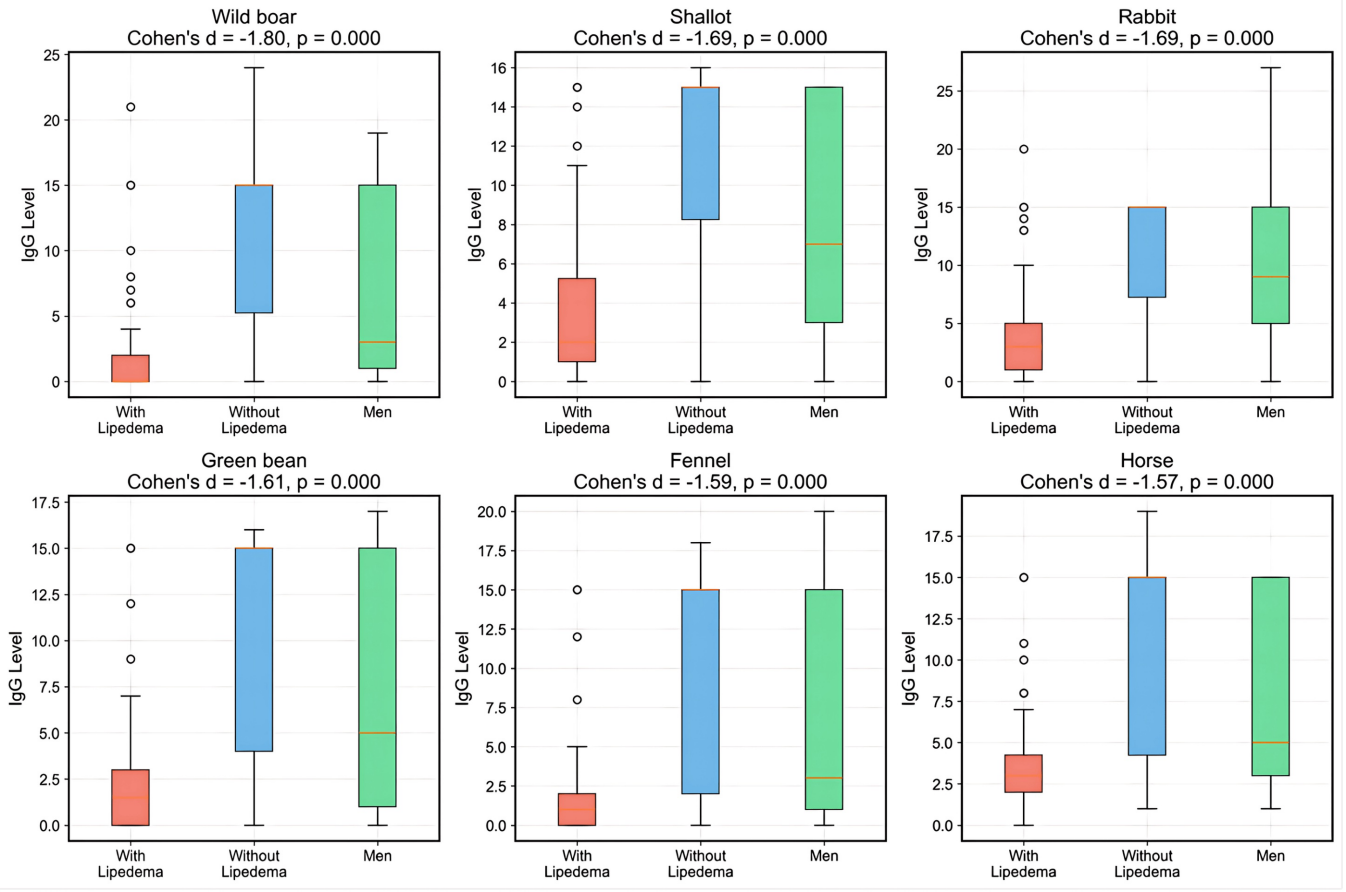
IgG distributions for the six antigens with the largest group differences Box plots of IgG reactivity (arbitrary units) for wild boar, shallot, rabbit, green bean, fennel, and horse—the antigens with the largest effect sizes (Cohen’s d) between women with lipedema (red), without lipedema (blue), and men (green). Boxes show median and IQR; whiskers denote 1.5×IQR; circles are outliers. For all six foods, IgG concentrations are significantly lower in the lipedema group (p < 0.001), with Cohen’s d ≈ −1.50 to −1.81, indicating strong discriminatory potential; the most pronounced differences are observed for wild boar, shallot, and rabbit.

The foods with the largest effect sizes (Cohen's d) were predominantly wild game meats and vegetables, including wild boar with d = -1.805 (p < 0.001), shallot with d = -1.694 (p < 0.001), rabbit with d = -1.685 (p < 0.001), green bean with d = -1.613 (p < 0.001), and fennel with d = -1.585 (p < 0.001). Representative distributions for the top six antigens by effect size are shown in Figure 5.

Combined diagnostic model

**Figure 6:**
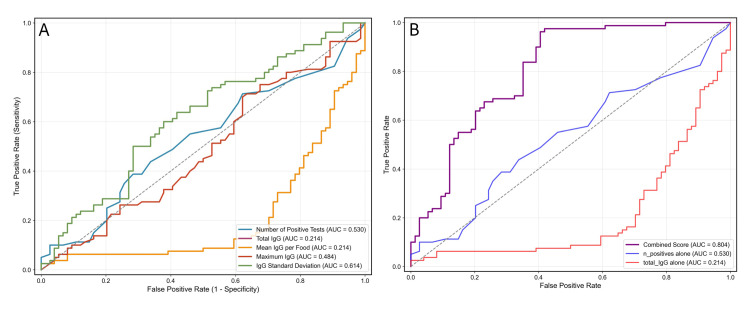
ROC curves for individual IgG metrics and a combined classification score (A) Receiver operating characteristic (ROC) curves for single-variable metrics: number of positive tests (AUC = 0.530), total IgG (AUC = 0.214), mean IgG per food (AUC = 0.214), maximum IgG (AUC = 0.484), and IgG standard deviation (AUC = 0.614). AUCs < 0.5 indicate an inverse association (lower values in lipedema). The dashed diagonal is the no-discrimination reference (AUC = 0.5). (B) ROC curve for the combined classification score (AUC = 0.804; cross-validated AUC = 0.771 ± 0.141) compared with the best single predictors (number of positives, AUC = 0.530; total IgG, AUC = 0.214). The combined score outperforms any individual metric.

A logistic regression model combining multiple IgG parameters achieved superior diagnostic performance with an AUC of 0.804 (95% CI: 0.736-0.872). The model included the number of positive tests (standardized β = 0.959), total IgG (β = -0.771), mean IgG per food (β = -0.771), and maximum IgG value (β = -0.217). Diagnostic performance for individual IgG metrics is shown in Figure 6A, whereas the combined score markedly improves discrimination (Figure 6B).

Pattern analysis

Correlation Analysis

**Figure 7:**
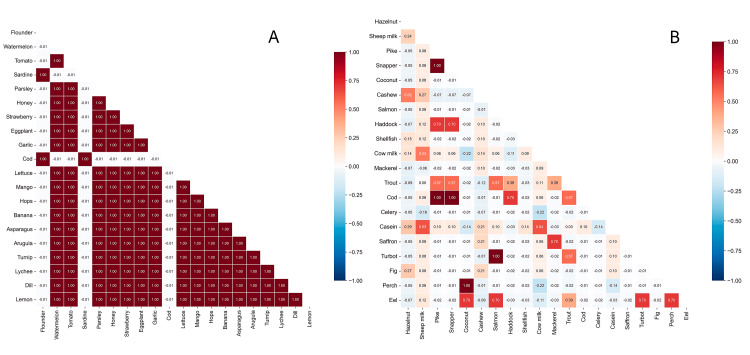
Pairwise correlations of IgG reactivity by group Triangular heatmaps of Pearson correlation coefficients (r) between IgG levels for selected food antigens in (A) women with lipedema and (B) women without lipedema. Cell values are the correlation coefficients; axes list antigens. Color scale reflects correlation strength (red = positive, blue = negative; −1 to +1). In the lipedema group (A), correlations are strong and widespread (many cells near r ≈ 1.0), especially among fruits and vegetables, indicating greater co-reactivity. In controls (B), correlations are more heterogeneous and generally weaker, with fewer high-r clusters.

Food reactivity patterns showed distinct correlation structures between groups. Women with lipedema demonstrated numerous perfect correlations (r = 1.0) among fruits and vegetables. Pairwise correlation structures are visualized in Figure 7.

Clustering Analysis

**Figure 8:**
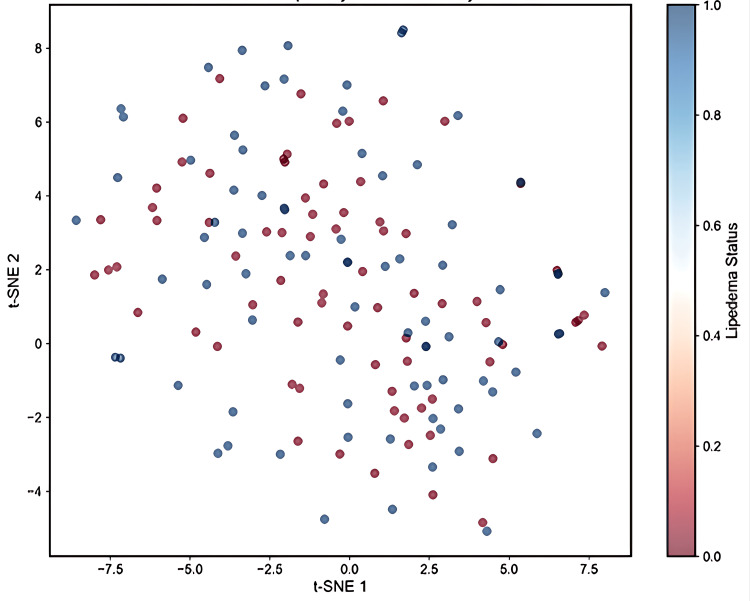
t-SNE visualization of IgG food-reactivity profiles in women Scatter plot of t-distributed stochastic neighbor embedding (t-SNE) applied to the 222-antigen IgG reactivity matrix. Each point represents one participant (woman); color encodes lipedema status (red = with lipedema, blue = without lipedema). Axes show t-SNE 1 and t-SNE 2 in arbitrary units. No distinct clustering or group separation is evident, indicating substantial overlap and heterogeneity in IgG response patterns between groups.

Dimensionality reduction techniques (PCA and t-SNE) revealed no clear separation between 80 women with and 74 without lipedema based solely on food reactivity patterns, indicating the complexity of the immune alterations. The lack of clear separation based on reactivity profiles is illustrated in Figure 8.

Food Category Analysis

**Figure 9:**
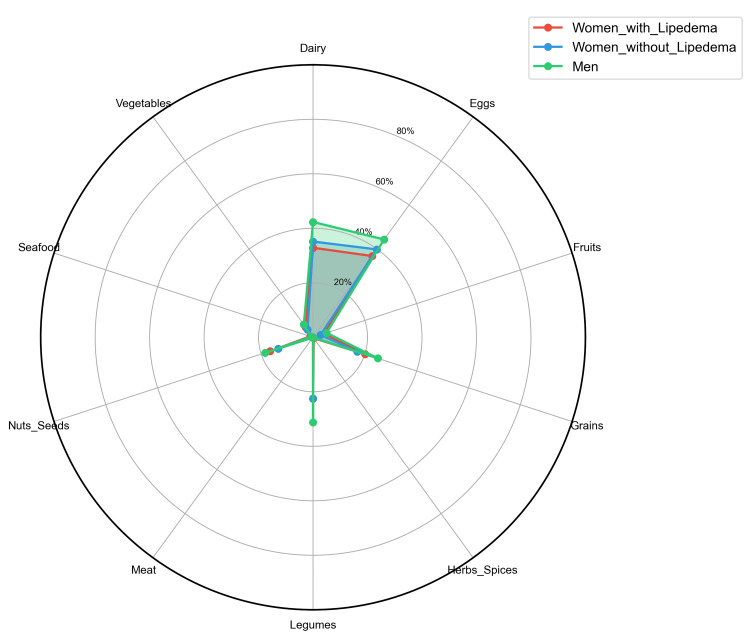
Food category IgG reactivity profile by group Radar chart displaying the average prevalence of positive IgG reactivity (%) across food categories for women with lipedema, women without lipedema, and men. All groups show the highest reactivity to dairy and eggs and low reactivity to meats, herbs/spices, and seafood. The overall similarity of patterns suggests shared exposure or immunogenicity at the category level, despite quantitative differences at the individual antigen level.

When grouped by food categories, all groups showed highest reactivity to dairy (32.9-42.3%) and eggs (36.9-44.4%), with minimal reactivity to meats (<0.1%) and herbs/spices (<0.4%). Category-level reactivity patterns are summarized in Figure 9.

Investigation of the IgG paradox

**Figure 10:**
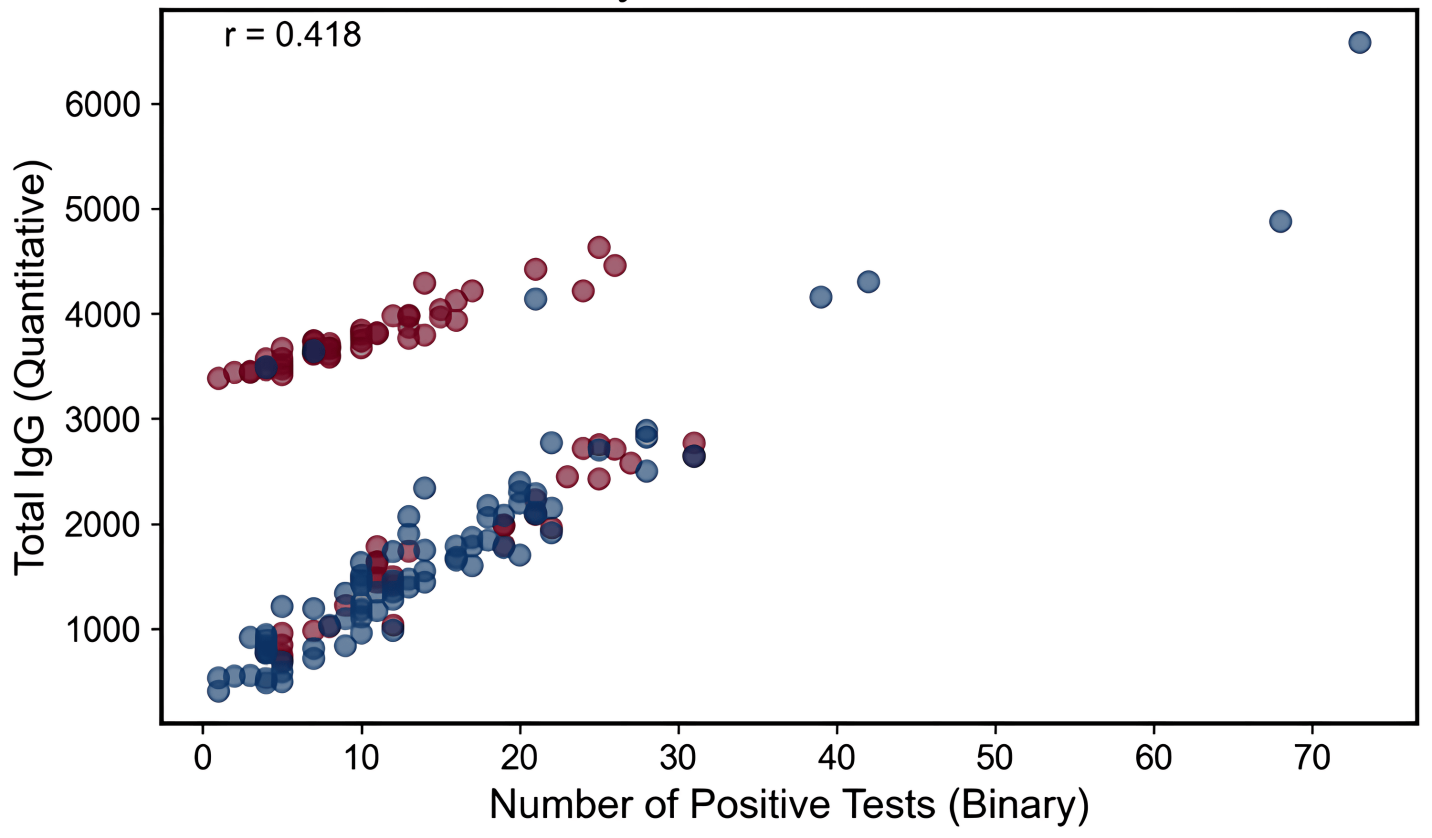
Relationship between the number of positive IgG tests and total IgG Scatter plot for all participants showing number of positive IgG food tests (x-axis, binary count) versus total IgG (y-axis, arbitrary units). Points are colored by status (red = with lipedema, blue = without). A moderate positive correlation is observed (r = 0.418), indicating that higher total IgG tends to accompany more positive tests, though with substantial dispersion (i.e., the relationship is not strictly linear).

To understand the paradoxical finding of more positive tests but lower IgG levels, we examined the relationship between qualitative and quantitative results. The correlation between the number of positive tests and total IgG was only moderate (r = 0.418), suggesting different underlying mechanisms. The relationship between the number of positive tests and total IgG is shown in Figure 10.

**Figure 11:**
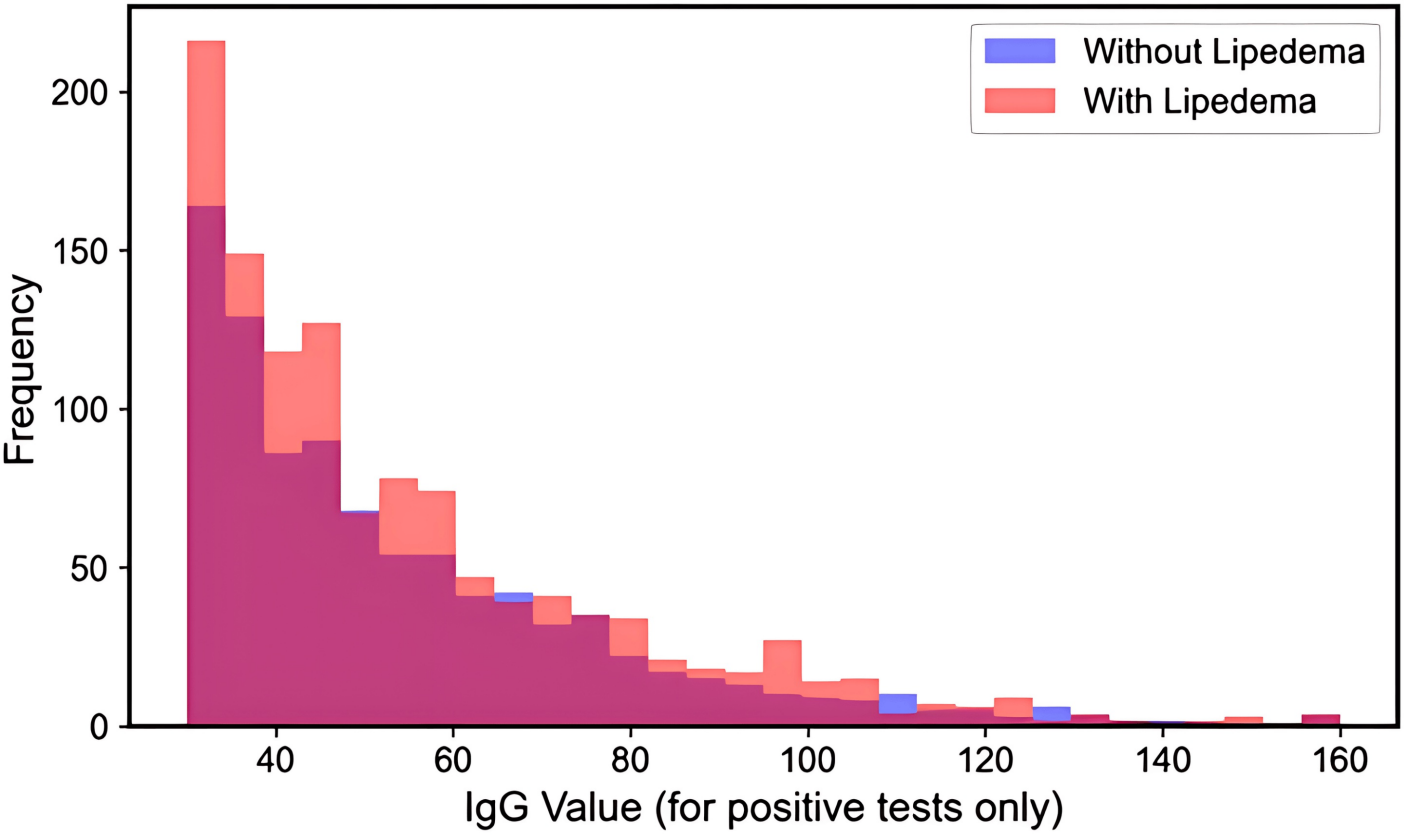
Distribution of IgG values for positive tests only Overlaid histograms of IgG concentration (arbitrary units) for tests classified as positive in women with lipedema (red) and women without lipedema (blue). The x-axis shows the IgG value; the y-axis shows frequency. The lipedema distribution is left-shifted, indicating more low-intensity positives despite a higher count of positives, consistent with the paradoxical pattern observed in this cohort.

Analysis of IgG intensity per positive test revealed that women with lipedema had markedly lower mean IgG values for their positive tests (118.1 ± 73.4 vs 236.1 ± 215.9, p < 0.001). The distribution of IgG intensity for positive tests only is presented in Figure 11.

## Discussion

This study presents the first comprehensive analysis of IgG food sensitivity patterns in lipedema, revealing a paradoxical immunological profile that challenges conventional understanding of food reactivity in chronic inflammatory conditions. This study identified a paradoxical IgG profile in lipedema-more reactivity, less antibody [16-18]. Our findings demonstrate that women with lipedema exhibit a unique pattern of increased food sensitivity frequency coupled with decreased IgG antibody production, suggesting complex alterations in immune regulation.

The central paradox

A key and unexpected finding was the apparent contradiction in IgG responses: despite showing a higher mean number of positive food reactions (14.8 vs 12.6), these patients demonstrated significantly lower total IgG levels (1747 vs 2975, p < 0.001) compared to controls. This paradox was consistent across 79.7% of individual food antigens tested, with effect sizes exceeding 1.5 for many foods. This pattern suggests that lipedema may be associated with a potentially distinct or dysregulated immune response to food antigens, characterized by increased sensitivity but diminished antibody production.

This paradoxical finding has several potential explanations. First, chronic inflammation in lipedema may lead to adaptive immunosuppression, where prolonged inflammatory stimulation results in decreased IgG production capacity [16-18]. Second, there may be a shift in IgG subclass distribution, with a potential transition from pro-inflammatory IgG1 and IgG3 to the more tolerogenic IgG4 subclass, which could explain the lower total IgG levels despite increased reactivity frequency [8]. Third, B cells in lipedema patients may exhibit lower activation thresholds, becoming activated by lower antigen concentrations but producing less antibody per cell. Fourth, increased IgG clearance or consumption in immune complexes could account for lower circulating levels [8]. Finally, dietary monotony in lipedema patients may result in repeated exposure to a limited range of foods, potentially inducing oral tolerance mechanisms that suppress IgG production while maintaining low-level reactivity [8,20-22].

Food-specific patterns

The analysis revealed consistent patterns of high reactivity across all groups to certain foods, particularly barley (>93%), peas (>85%), egg white (>67%), cow milk (>76%), and kola nut (>71%). These foods represent common dietary staples and known allergens, suggesting that some degree of IgG reactivity to these antigens may be physiological. However, specific foods showed significant differences between groups. Sunflower seed (23.75% vs 8.11%, p=0.02) and aloe vera (12.50% vs 2.70%, p=0.05) demonstrated higher prevalence in lipedema patients, potentially indicating unique dietary exposures or reflecting idiosyncratic immune responses specific to the lipedema phenotype.

The quantitative analysis revealed even more striking differences, with wild game meats and certain vegetables showing the largest effect sizes [21]. The consistently lower IgG levels to these foods in lipedema patients, despite similar or higher prevalence of positivity, reinforces the concept of altered immune regulation rather than simple increased reactivity [16-18].

Pattern recognition and clustering

Our dimensionality reduction analyses (PCA and t-SNE) failed to demonstrate clear separation between women with and without lipedema based solely on food reactivity patterns. This finding suggests that the immune alterations in lipedema are quantitative rather than qualitative, affecting the magnitude of responses rather than creating entirely distinct reactivity profiles. The lack of clustering also indicates that IgG food sensitivity patterns alone are insufficient for lipedema classification, emphasizing the need for integrated diagnostic approaches.

Subgroup heterogeneity analysis

The significant difference observed only in the low reactivity tertile (p=0.040), with lipedema patients paradoxically showing fewer positive tests than controls in this subgroup, suggests immunological heterogeneity within the lipedema population. This heterogeneity may explain why overall comparisons showed no significance (p=0.186), as combining immunologically distinct subgroups could dilute real differences. Proper stratification of lipedema patients by immune phenotype might reveal stronger statistical associations and further highlight the paradoxical relationship between reduced reactivity frequency in certain subsets and consistently diminished total IgG production across all patients.

Clinical implications

The development of a combined diagnostic model incorporating multiple IgG parameters achieved an AUC of 0.804, representing good discriminative ability. Such a model may not replace clinical diagnosis but could support screening or subgroup identification in research and practice. The strong negative weighting of total IgG levels in the model (-0.771) alongside positive weighting of the number of positive tests (0.959) mathematically captures the paradoxical pattern we observed.

These findings support the hypothesis of intestinal barrier dysfunction in lipedema. The increased frequency of food reactions suggests enhanced intestinal permeability, allowing greater antigen exposure, while the diminished IgG response indicates dysregulated immune processing of these antigens. This dual alteration may not only underlie some clinical features of lipedema but also serve as a basis for developing personalized interventions, particularly those targeting diet or mucosal immune modulation [16-18].

This immunological signature further suggests that IgG-based immune profiling could help tailor dietary recommendations or distinguish clinically relevant subgroups within the heterogeneous lipedema population. Encouraging dietary diversity may help rebalance immune tolerance mechanisms in lipedema patients, potentially reducing subclinical food reactivity and chronic inflammation [8,15].

Mechanistic considerations

These immune alterations may stem from dysregulated cellular signaling pathways involving B-cell activation, antibody class switching, and chronic antigen exposure. Such changes likely reflect the downstream effects of prolonged immune stimulation in the lipedema microenvironment. Adipose tissue in lipedema is known to be pro-inflammatory, and our findings suggest this inflammation extends to altered systemic immune responses. The pattern of increased sensitivity with decreased antibody production is reminiscent of immune exhaustion seen in chronic inflammatory conditions, where persistent stimulation leads to functional impairment of immune cells [8,16,18].

The possibility of dietary monotony contributing to these patterns deserves further investigation. If lipedema patients consume less varied diets, perhaps due to food aversions or attempts at dietary management, this could lead to the observed pattern through mechanisms of oral tolerance and immune regulation.

Consistent with the endotoxin-complement cascade hypothesis proposed by Kruglikov and Scherer [20], which implicates low-grade endotoxemia and dysregulation of the alternative complement pathway as key drivers in lipedema pathophysiology, specifically, the paradoxical pattern observed in our study, characterized by increased frequency of positive IgG food reactions coupled with reduced total IgG levels, may reflect a state of chronic immune activation induced by LPS exposure in gluteofemoral white adipose tissue (gfWAT). LPS is known to activate both TLR4 and the ACP, leading to inflammasome activation and immune remodeling. The suppression of CFD observed in lipedema may prevent full ACP activation, resulting in sublytic MAC production, NLRP3 inflammasome priming, and downstream alterations in immune signaling without overt cytotoxicity [20]. This mechanism could explain the presence of persistent, low-intensity food antigen reactivity in the absence of robust antibody production. Furthermore, our results support the hypothesis that increased intestinal permeability (leaky gut) may underlie the antigenic load driving this immune state and suggest that dietary modulation or endotoxin management may hold therapeutic potential in lipedema.

Limitations

Several limitations should be considered when interpreting these results. First, the retrospective design and convenience sampling may introduce systematic bias. Patients with lipedema may be more likely to seek IgG food sensitivity testing due to chronic symptoms or prior dissatisfaction with conventional treatments, potentially inflating observed reactivity patterns.

Second, the absence of essential clinical data limits the interpretability and generalizability of the findings. Due to the anonymized nature of the dataset, we were unable to collect information on lipedema stage or severity, disease duration, BMI and body composition, current medications, or dietary habits. These clinical phenotypes may significantly influence immune responses and food sensitivity profiles. The lack of this information constitutes a major limitation, as it precludes adjustment for key confounding variables.

Finally, the commercial IgG panel used may have varying sensitivity and specificity across different food antigens, and subclass-specific IgG data were not available. We also did not assess gut microbiota composition, which may influence mucosal immunity and food antigen tolerance. Additionally, some foods that tested positive may reflect immunologic cross-reactivity with other foods that share homologous epitopes (pan-allergens), rather than true reactivity to the specific item [21] (see Table 5). Moreover, some women with lipedema may be adhering to restrictive or targeted diets (e.g., ketogenic or elimination protocols), which can alter recent exposure to specific foods and bias both the number of positives and the measured IgG intensities, typically lowering values due to reduced antigen exposure [22]. In line with these limitations, patient-education resources outlining personalized elimination-rechallenge protocols and symptom diaries can support adjunctive clinical management [23].

These mechanistic explanations are plausible but remain unproven within the constraints of our retrospective dataset. Future studies should include detailed clinical phenotyping and longitudinal designs to clarify the temporal relationship between immune profiles and disease progression, ideally integrating dietary, metabolic, and microbiome data to develop more personalized diagnostic and therapeutic strategies.

Future directions

Future research should prioritize prospective studies with detailed clinical phenotyping of patients with lipedema, including disease stage, duration, BMI, body composition, medication use, and dietary history. Such data are essential to contextualize IgG reactivity patterns and to control for potential confounders influencing immune responses.

Additionally, investigation of IgG subclass distribution, particularly IgG4 [8], may offer deeper mechanistic insights into immune tolerance versus activation. Integrating food antigen profiling with gut microbiota composition, intestinal permeability markers [16-18], and metabolomic or transcriptomic data could help clarify the immunopathology underlying lipedema and identify subgroups with distinct inflammatory or metabolic profiles.

Exploring the longitudinal evolution of IgG reactivity patterns, their relationship to symptom burden and treatment response, and the impact of dietary diversity or immune-targeted interventions may inform personalized management strategies and improve clinical outcomes [22].

## Conclusions

In this retrospective cohort of women, we observed a paradoxical IgG signature in lipedema: a modestly higher frequency of food-specific positives despite markedly lower total/mean IgG levels, consistent with mucosal immune dysregulation. Individually, summary IgG metrics and the count of binary positives showed limited discrimination (AUC 0.21-0.53), whereas a combined score integrating frequency and intensity achieved good in-sample performance (AUC = 0.804; internal cross-validated AUC = 0.771 ± 0.141), supporting IgG profiling as a complementary, not standalone, candidate biomarker for subgroup identification and hypothesis generation.

Mechanistically, these findings are compatible with a barrier-centric model in which increased intestinal permeability and chronic antigen/LPS exposure remodel systemic immunity (including complement dynamics) and favor low-intensity, widespread reactivity. Viewed together, these patterns raise the possibility that lipedema is not a primary disease of adipose tissue but an adipose response to upstream systemic processes; this adipose-response hypothesis is provisional and requires external validation. Prospective studies should integrate IgG subclasses, barrier markers, diet exposures (including gluten withdrawal/re-challenge), and HLA-DQ2/DQ8 with external validation to test causality and therapeutic relevance.

## Appendices

Food-specific IgG positives can arise from immunologic cross-reactivity between taxonomically related foods (pan-allergens) or even non-food sources (see Table 5) [21]. Several high-prevalence or discriminative items in our dataset map to known cross-reactive families (e.g., barley→gluten cereals; milk/casein→other dairy species; egg white→other avian eggs; pea/green/white/red beans→other legumes; sunflower seed→Asteraceae; shallot→Allium; fennel→Apiaceae; wild boar→pork; rabbit→hare; razor clam→other mollusks). In our setting, pea protein isolate is widely used in ultra-processed foods, so “pea” positivity may reflect ubiquitous exposure rather than a specific intolerance. These mechanisms can inflate the number of distinct foods flagged per person without indicating independent sensitizations and should temper causal interpretations of individual foods.

**Table 7:** Cross-reactivity considerations for key foods highlighted in this study Examples are illustrative, not exhaustive; cross-reactants summarized from institutional cross-reactivity reference material. Index foods correspond to items with high prevalence or large effect sizes in the present study. Interpretation should be integrated with diet history and clinical context.

Index Food (This Study)	Likely Cross-Reactants (Examples)	Mechanistic Note (Typical Shared Proteins)	Practical Interpretation
Barley	Wheat, rye, spelt (gluten cereals)	Prolamins (hordein/gliadin/secalin)	Consider gluten-wide exposure when interpreting a single-item positive.
Cow/Sheep/Goat milk; Casein	Buffalo/goat/sheep/cow milk (cross-dairy)	Caseins & whey proteins	A single dairy positive may reflect dairy-wide cross-reactivity.
Egg white	Other avian eggs (quail, duck, etc.)	Ovomucoid/ovalbumin	Positivity may generalize to other eggs.
Pea	Chickpea, lentil, peanut, soy (legumes)	Storage proteins (vicilins/legumins)	May reflect legume cluster and/or high exposure via ultra-processed foods.
Green/White/Red beans	Other beans/legumes	Storage proteins	Interpretable as family-level legume reactivity.
Sunflower seed	Artichoke, chamomile, chicory, dandelion, lettuce, tarragon (Asteraceae)	Pan-allergens in Asteraceae	Co-positivity within Asteraceae is plausible.
Shallot	Onion, garlic, leek, chive (Allium)	Allium proteins (e.g., alliinase)	Treat as Allium-family pattern rather than an isolated item.
Fennel	Celery, carrot, cumin, dill, parsley, coriander (Apiaceae)	Apiaceae pan-allergens	Cluster-level interpretation recommended.
Wild boar	Pork	Serum albumins/heat-stable proteins	Meat species cross-reactivity possible.
Rabbit	Hare	Muscle/serum proteins	May reflect game-meat cluster.
Razor clam	Clam, mussel, oyster, scallop (mollusks)	Tropomyosin & related	Consider mollusk-wide cross-reactivity.
Kola nut	Cocoa, coffee	Seed proteins; reported cross-links	May indicate stimulant seed exposure cluster.
Cauliflower	Broccoli, cabbage, Brussels sprouts, mustard/canola (Brassicaceae)	Crucifer proteins	Reads as Brassicaceae pattern.
Aloe vera	— (none clearly established)	Not well-defined	Positivity may reflect exposure/assay behavior more than family cross-reactivity.
